# Research note: Bioaccumulation of β-*N*-methylamino-L-alanine and 2,4-diaminobutyric acid in chicken eggs

**DOI:** 10.1016/j.psj.2026.107682

**Published:** 2026-08-22

**Authors:** Sea-Yong Kim, Elin Lindehoff, Catherine Legrand, Ji Yoon Kim, Sara Rydberg

**Affiliations:** a Department of Biology, Chungbuk National University, Cheongju, Chungbuk, 28644, Republic of Korea; b Centre of Ecology and Evolution and Microbial Model Systems, Department of Biology and Environmental Science, Linnaeus University, 39231, Kalmar, Sweden; c School of Education and Communication, Jönköping University, Jönköping, Sweden; d Department of Bioscience and Biotechnology, Chungnam National University, Daejeon, 34134, Republic of Korea; e Department of Ecology, Environment and Plant Sciences, Stockholm University, SE 10691, Stockholm, Sweden

**Keywords:** Neurotoxins, Bioaccumulation, Egg

## Abstract

β-N-methylamino-L-alanine (BMAA) and its structural isomer 2,4-diaminobutyric acid (DAB) are non-proteinogenic amino acids known to cause neurotoxicity. Although both compounds have been detected in a range of organisms, their presence in chicken eggs has not been examined. We analyzed egg white and yolk from 30 Swedish eggs obtained from a small-scale producer to assess the bioaccumulation of BMAA and DAB. Both toxins were detected predominantly in egg white. Mean concentrations in egg white were 5.6 ± 3.9 ng g^–1^ dry weight (DW) for BMAA and 7.1 ± 4.6 ng g^–1^ DW for DAB, compared with 0.7 ± 1.6 ng g^–1^ DW and 0.9 ± 0.7 ng g^–1^ DW in egg yolk, respectively. The corresponding concentrations of BMAA and DAB on a wet weight (WW) basis were 0.90 ± 0.62 ng g^–1^ WW and 1.14 ± 0.74 ng g^–1^ WW in egg white, and 0.34 ± 0.77 ng g^–1^ WW and 0.43 ± 0.34 ng g^–1^ WW in egg yolk, respectively. These findings indicate that chicken eggs from small-scale production systems may represent a previously underrecognized exposure route to BMAA and DAB, highlighting the need for broader surveillance across large-scale commercial egg production systems to confirm whether chicken eggs are a potential source of dietary exposure.

## Introduction

The non-proteinogenic amino acid β-N-methylamino-L-alanine (BMAA) and its structural isomer 2,4-diaminobutyric acid (DAB) exert neurotoxic effects primarily through excitotoxicity (Weiss et al., 1989), with additional mechanisms such as protein misincorporation and oxidative stress also proposed (Downing and van Onselen, 2021).

The bioaccumulation and biomagnification of BMAA were first reported in the terrestrial food web of Guam during the early 2000s (Cox et al., 2003) and have since been reported in diverse aquatic and agro-aqua environments (Réveillon et al., 2016; Kim and Rydberg, 2020; Wang et al., 2021). In poultry, BMAA has been detected in muscle, liver, brain, and eye tissues of chickens raised in the agro-aqua systems (Kim and Rydberg, 2020). Vertical transfer of BMAA has also been demonstrated in Japanese quail, where the toxin accumulated predominantly in egg yolk (Andersson et al., 2018). However, no study has examined whether DAB can be vertically transferred in any species, nor whether either toxin occurs in chicken eggs intended for humans. Given the documented accumulation of BMAA in poultry tissues as well as quail eggs (Andersson et al., 2018; Kim and Rydberg, 2020), we hypothesized that BMAA and DAB may also be present in chicken eggs. The objective of the present study was therefore to determine the occurrence and distribution of BMAA and DAB in Swedish chicken eggs.

## Materials and methods

### Sample collection

Hens of the Lohmann white breed were sourced from a local scale egg producer in Sweden. Eggs (*n* = 30) were collected under controlled conditions, where the egg-laying hens were monitored by a bird-specialist veterinarian throughout the production period. Hens were kept in an indoor housing area of 2 × 5 m (10 m²), accessible at all times, with access to an outdoor pen of 2 × 12 m (24 m²) during daylight hours. Feed (organic layer feed, Swedish Agro AB) was supplied ad libitum via a feed dispenser and supplemented with a calcium source consisting of crushed mussel shell/meal (*Mytilus* spp.). The final delivered feed contained 91.1 % dry matter. Egg white and yolk were separated after collection and stored at –80°C until extraction and analysis.

### The analysis of neurotoxins

Samples were lyophilized using a CoolSafe freeze dryer (SCANVAC, Stockholm, Sweden) with the condenser temperature set at −110 °C. Each biological replicate was analyzed in four technical replicates. The extraction of the toxins BMAA and DAB followed the protocol described by Kim and Rydberg (2020). Samples were diluted using 20 mM HCl to optimize the ratio of protein to derivatization agent. The diluted samples were derivatized with a WAT052880 AccQ-Tag kit (Waters, Milford, MA, USA).

Quantification was performed using an Acquity UPLC system coupled to a Xevo TQ MS system (Waters). Separation was achieved on an AccQ-Tag Ultra C18 column (100 × 2.1 mm, 1.7 μm particle size, Waters) with a binary mobile phase (A: 0.01 % formic acid in water; B: 0.01 % formic acid in methanol) delivered at a flow rate of 0.5 mL min^−1^. Analytes were detected in positive ion mode using electrospray ionization and the selected reaction monitoring (SRM) scan mode with the transitions of 459.1 > 119.08 for all analytes, 459.1 > 188.1 for DAB diagnostic fragment, and 459.1 > 258.09 for BMAA diagnostic fragment. The instrument parameters were 30 V for cone voltage, 150°C for source temperature, 550°C for desolvation temperature, 20 L h^–1^ for cone gas flow, 1000 L h^–1^ for desolvation gas flow, 0.15 mL min^–1^ for collision gas flow, and 26 V for collision energy. Chromatographic data were processed using MassLynx V4.1 software (Waters, Stockholm, Sweden) and differences between white and yolk concentrations were assessed using Welch’s *t-test*.

### Quantification of neurotoxins and quality controls

All egg white and yolk samples obtained from the 30 eggs were screened for BMAA and DAB before quantification. Samples in which neither BMAA nor DAB was detected were selected and used as blank matrices. No analyte signals corresponding to the retention times and diagnostic transitions of either BMAA or DAB were observed in the selected blank matrices. These matrices were used to prepare calibration curves and to perform recovery and precision tests.

BMAA and DAB standards were purchased from Sigma-Aldrich (St. Louis, MO, USA). Calibration standards for both BMAA and DAB were prepared in egg white and yolk matrices at six concentrations ranging from 0.3 to 60 ng mL^−1^. The limit of quantification (LOQ) was determined separately for each toxin and matrix, based on a signal-to-noise (S/N) ratio of five. The LOQs for BMAA were 0.5 ng mL^−1^ in egg white and 0.3 ng mL^−1^ in yolk, whereas DAB showed an LOQ of 0.5 ng mL^−1^ in both matrices. For the recovery and the intra- and inter-day precision tests, blank matrices were spiked with BMAA and DAB at 5 ng mL^−1^ (*n* = 3), and these spiked matrices also served as positive controls.

## Results and discussion

The resulting calibration plots exhibited the linearity with R^2^ > 0.99. Mean recovery for BMAA ranged from 87 to 92 % (white: 92±5 %, yolk: 87±5 %), and DAB recovery ranged from 90 to 100 % (white: 90±4 %, yolk: 100±5 %) (Table 1). The intra- and inter-day precision values were all below 10 %.

**Table 1:** The results of the recovery test.

Toxins	Sample type	Recovery (%)
**BMAA**	White	92±5
	Yolk	87±5
**DAB**	White	90±4
	Yolk	100±5

**Fig. 1:**
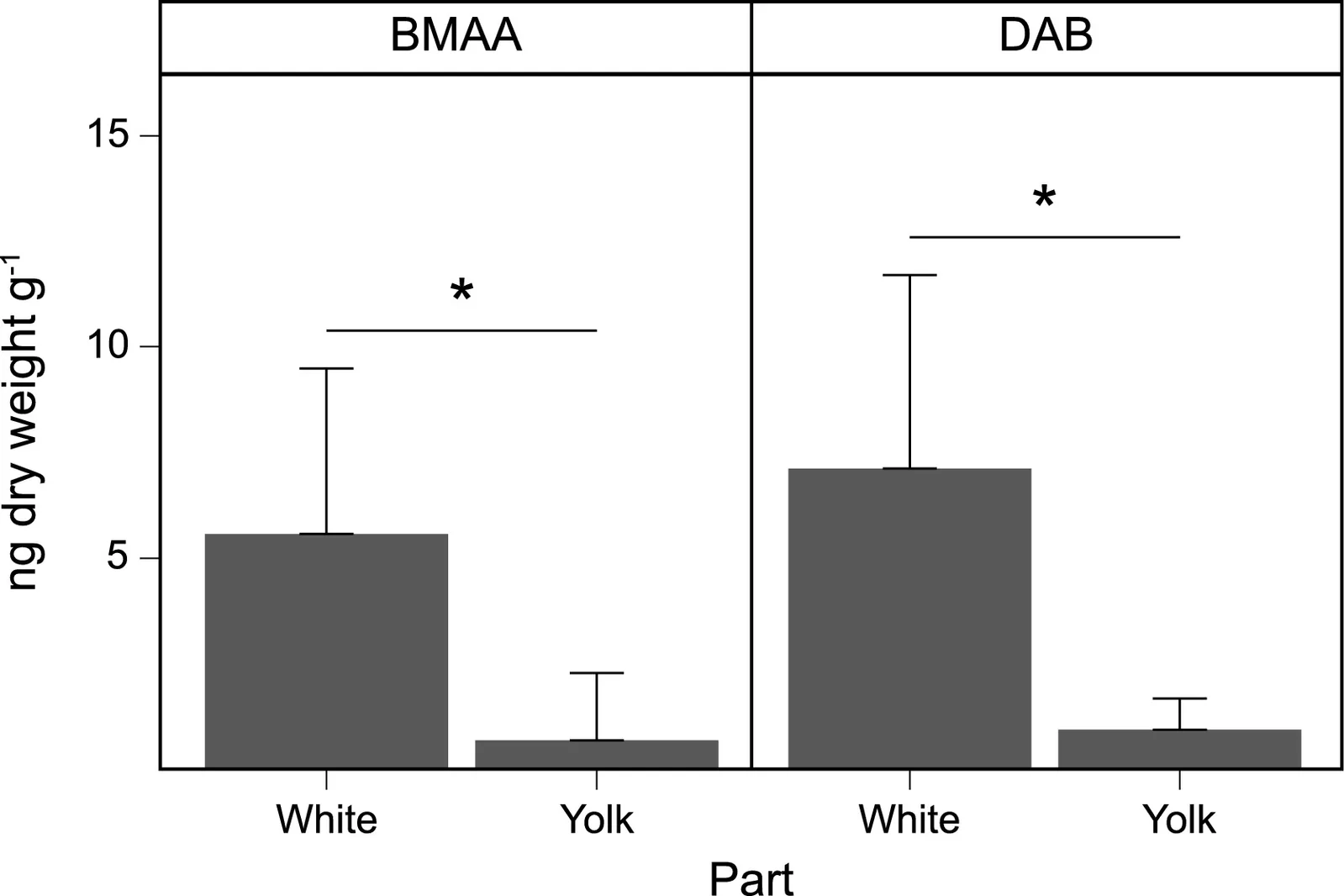
The concentration of BMAA and DAB in egg white and yolk. The concentrations are expressed as mean ± standard deviation (ng dry weight (DW) g^–1^, *n* = 30). Significant differences (*p* < 0.05) between the concentrations of white and yolk are presented by asterisks. The *t*-test was used for two-group comparison.

BMAA was detected in 27 of 30 eggs. Among these 27 eggs, BMAA was detected in the egg white of 26 eggs and in the yolk of four eggs. Three eggs showed BMAA in both egg white and yolk, while one egg showed BMAA only in the yolk. Concentrations were significantly (p < 0.05) higher in egg white than in yolk, with a concentration of 5.6 ± 3.9 ng BMAA g^–1^ DW (0.90 ± 0.62 ng g^–1^ WW) and 0.7 ± 1.6 ng BMAA g^–1^ DW (0.34 ± 0.77 ng g^–1^ WW), respectively (Fig. 1).

DAB was detected in 29 of 30 eggs. In all 29 positive eggs, DAB was detected in both egg white and yolk, and concentrations were significantly higher (p < 0.05) in egg white than in yolk. The concentration of DAB was 7.1 ± 4.6 ng DAB g^–1^ DW (1.14 ± 0.74 ng g^–1^ WW) in the egg white (*n* = 29 out of 29) and 0.9 ± 0.7 ng DAB g^–1^ DW (0.43 ± 0.34 ng g^–1^ WW) in the yolk (*n* = 29 out of 29) (Fig. 1).

Both toxins were detected at higher concentrations in egg white than in yolk; the concentrations in egg white were eight times higher on a DW basis and 2.6 times higher on a WW basis. No other studies have examined the accumulation and distribution of DAB in bird eggs yet. The BMAA pattern observed in the present study contrasts with findings in Japanese quail, where BMAA accumulated predominantly in the yolk following exposure to 34 mg carboxyl-^14^C/methyl-^14^C BMAA kg^–1^ body weight (Andersson et al., 2018). In that study, radioactivity levels were 3 to 13 times higher in the yolk than in the albumen (Andersson et al., 2018). These species-specific differences suggest that toxin partitioning within eggs may depend on physiological or biochemical factors such as amino acid composition, mineral distribution or protein affinity.

Most nutrients are preferentially allocated in egg yolk, but only three out of 12 minerals and one out of 12 vitamins occur in relatively higher amounts in egg white than in the yolk: sodium, chlorine, and potassium for the minerals, and B3 (niacin) for the vitamins (Nys and Sauveur, 2004; Seuss-Baum, 2007). In addition, one study (Kudre et al., 2018) compared amino acid composition in the eggs of Japanese quail and Leghorn white hens. Of the 17 amino acids analyzed, two were present at higher concentrations in the egg white of Leghorn white hens compared with the quail, while being lower in the yolk. Specifically, the proportion of histidine in egg white was 1.38:2.56 (quail:Leghorn) and 1.22:1.09 in yolk, and the proportion of isoleucine was 6.75:6.89 in egg white and 6.89:5.84 in yolk, respectively. These findings indicate that further studies are needed to clarify the affinity of BMAA and DAB for different egg components in poultry.

The present study shows that BMAA and DAB can accumulate in chicken eggs, suggesting that chicken eggs may act as a route of human exposure to these neurotoxins. However, the results are derived from eggs collected within a small-scale production setting, where other environmental exposures were not characterized, and therefore should not be broadly generalized. Additional studies using eggs from large-scale commercial production systems are needed to validate these findings.

**Ethics declaration**

**Animal subject**

Ethics committee approval was not required. The study analyzed eggs collected from laying hens under routine production conditions. No experimental treatment was performed on the hens. An unfertilized egg is not considered an animal in research ethics.

## CRediT authorship contribution statement

**Sea-Yong Kim:** Writing – review & editing, Writing – original draft, Validation, Methodology, Investigation, Formal analysis, Conceptualization. **Elin Lindehoff:** Writing – review & editing, Validation, Data curation. **Catherine Legrand:** Writing – review & editing. **Ji Yoon Kim:** Writing – review & editing, Validation. **Sara Rydberg:** Writing – review & editing.

## Disclosures

The authors declare no conflicts of interest.
